# A succinylation switch to maligancy: SUCLG1, mitochondrial transcription and leukemia

**DOI:** 10.1038/s44318-024-00116-2

**Published:** 2024-05-09

**Authors:** Laura Guerrero, Panagiotis Ntziachristos

**Affiliations:** 1 Leukemia Therapy Resistance Unit, Department of Biomolecular Medicine, Ghent University, Ghent, Belgium; 2 Center for Medical Genetics, Ghent University and University Hospital, Ghent, Belgium; 3 Cancer Research Institute Ghent (CRIG), Ghent, Belgium

**Keywords:** Cancer, Haematology, Metabolism

## Abstract

Recent work uncovers a role of mutant FLT3-driven Krebs cycle enzyme SUCLG1 in AML, derepressing mitochondrial gene expression and mass.

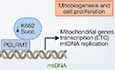

## Teaser

The cellular consequences of activating FLT3 mutations in blood cancers are poorly understood. In this issue, Yan et al (2024) demonstrate that FLT3 activation promotes mitobiogenesis and leukemia progression through a posttranslational control mechanism, linking cell metabolism with derepressed mitochondrial transcription and function.

Acute myeloid leukemia (AML) is the most common type of hematologic cancer in adults. Despite the progress to date in the development of anti-cancer drugs, refractory and relapsed disease represent very challenging aspects in AML. The current focus of research efforts in the field is on understanding resistance to therapy and relapse mechanisms via the identification of mutations involved (Dohner et al, 2015). The mutated and constitutively activated FMS-like tyrosine kinase three (FLT3) is one of the most common genetic aberrations in AML. FLT3 is a receptor tyrosine kinase that plays a pivotal role in the normal development of hematopoietic stem cells and progenitor cells. FLT3 mutations occur in approximately 30% of all AML cases, with the internal tandem duplication (ITD, FLT3^IDT^) representing the most common type of FLT3 mutation. FLT3^IDT^ confers a poor prognosis in AML patients, as it promotes leukemic cell survival and proliferation through activation of RAS/MAPK, PI3K/AKT/mTOR and JAK/STAT5 pathways, among other mechanisms. FLT3 mutations, notably FLT3^IDT^, drive constitutive activation of FLT3 kinase and downstream pathways promoting proliferation, survival, and metabolism in AML, implicating mitochondrial biogenesis.

Besides their central bioenergetic functions, mitochondria participate in tumor metabolism, redox and calcium homeostasis, transcriptional regulation, and cell death. Central to mitochondria function is the tricarboxylic acid (TCA) cycle or Krebs cycle: It starts with the combination of acetyl-CoA, derived from carbohydrates, fats, and proteins, with oxaloacetate forming citrate, which is then further transformed, releasing carbon dioxide, the reducing agents NADH and FADH2, and directly generating ATP or GTP. The original acceptor molecule, oxaloacetate, is ultimately regenerated to start the process again, providing energy substrates for the electron transport chain. Mitochondria also regulate apoptosis primarily through the release of cytochrome c, initiating a cascade that activates caspases for cell death, governed by the balance between pro-apoptotic and anti-apoptotic Bcl-2 family proteins. Oncogenic *FLT3* mutations hijack mitochondrial biogenesis to promote AML growth. The mutant, hyperactive protein leads to metabolic reprogramming, enhancing glucose uptake and glycolysis, potentially boosting mitochondrial biogenesis to adapt to altered metabolism in leukemic cells (Gallipoli et al, 2018). Furthermore, activated FLT3 signaling increases reactive oxygen species (ROS) production, which may modulate mitochondrial biogenesis to satisfy energy demands and manage oxidative stress (Ju et al, 2017; Sallmyr et al, 2008).

**Figure 1:**
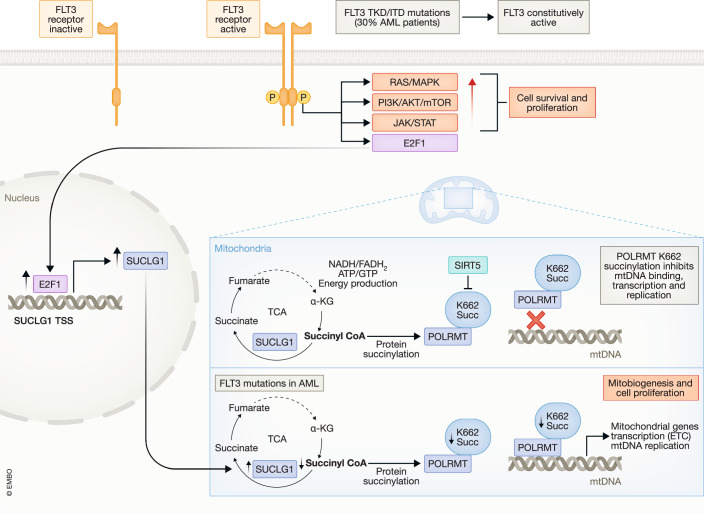
SUCLG1 controls POLRMT activity, mitochondrial gene transcription, and mitochondrial biogenesis in FLT3-mutant leukemia. Succinylation is a post-translational modification that attaches a succinyl group to lysine residues, significantly altering the protein’s mass, charge, and conformation, affecting protein function and stability. In the case of POLRMT, succinylation prevents its binding to DNA. FLT3 mutations trigger SUCLG1 upregulation and consequent reduction of POLRMT K662 succinylation, crucial for mtDNA transcription and replication. In turn, SIRT5 reverses POLRMT succinylation and helps restore POLRMT binding to DNA, thus maintaining the mitochondrial function. SIRT5 inhibition blocks mitochondrial transcription and is a potential therapeutic target in AML treatment.

Despite these insights, the direct impact of *FLT3* mutations on mitochondrial biogenesis remains to be fully understood, underscoring the need for further research. In this work, Yan et al, (2024), provide mechanistic and functional insights into how mutant FLT3 hijacks mitochondrial gene expression and biogenesis to promote AML growth and suggest therapeutic interventions (see Fig. 1). The authors uncovered that mutant FLT3 elevates the expression of *SUCLG1*, which codes for a TCA enzyme that converts succinyl-CoA to succinate, without affecting the levels of other TCA-related transcripts. Analyses of the Cancer Genome Atlas (TCGA) data showed a correlation between the expression of SUCLG1 and genes associated with the electron transport chain. Hyperactive FLT3 induces E2F1, which in turn transcriptionally controls SUCLG1. This finding was supported by discovering E2F1-binding motifs near the SUCLG1 gene and further confirmed through ChIP-qPCR experiments. Increased levels of SUCLG1 lead to decreased succinyl-CoA and increased succinate levels. The authors show that this succinyl-CoA decrease reduces succinylation of the mitochondrial polymerase POLRMT at lysine 622 (K622) and boosts its activity on the mitochondrial genome encoding 13 proteins critical for oxidative phosphorylation and related non-coding RNAs. Indeed, POLRMT succinylation is detrimental to POLRMT’s ability to bind mtDNA (Kuhl et al, 2016). To this end, SUCLG1 loss leads to reduced mitochondrial mass, diminished activity of all five electron transport chain complexes, increased ROS production, and decreased cell proliferation.

Mitochondria constitute promising targets for developing novel anticancer agents: in hematological malignancies, there is a strong relationship between cellular metabolism, mitochondrial bioenergetics, and cell proliferation. Increased mitochondrial biogenesis (mitobiogenesis) of tumor cells results in augmented ATP production and drug resistance. Therefore, targeting mitobiogenesis, or mitochondrial DNA replication and transcription, increases therapy efficacy (Barbato et al, 2020). Several compounds targeting mitochondrial metabolism in leukemia have been reported, such as ellagic acid, sodium dichloroacetate, acacetin, 2-methoxiestradiol, FK866, and venetoclax. Venetoclax is a selective BCL2 inhibitor that has received FDA approval for treating AML. However, drug resistance represents a heavy burden. A recent study using a genome-wide CRISPR/Cas9 screen in human AML interestingly identified that genes involved in mitochondrial organization and function participate in the acquisition of venetoclax resistance (Chen et al, 2019). The current authors (Yan et al, 2024) investigated the function of SIRT5, a NAD-dependent protein desuccinylase, and its potential as a therapeutic target. SIRT5 inhibition resulted in increased POLRMT succinylation, which in turn led to a reduction in mtDNA binding and the expression of mtDNA-encoded genes, suggesting that SIRT5 inhibitors can be used for targeting mitochondria biogenesis in the course of the treatment of FLT3-mutant AML.

The current work by Yan and colleagues, (2024) prompts us to pose new questions and discuss challenges: Initially, gaining insight into the array of E2F cofactors and targets within this system will be of interest, followed by evaluating interventions at the transcriptional level. While SUCLG1 inhibition increases POLRMT succinylation that reflects on mitochondrial transcription, there are additional potential functions of SUCLG1 in cell metabolism and proliferation that require further exploration. Investigating potential players (enzymes) controlling POLRMT succinylation in addition to SUCLG1 would be compelling. To ascertain broader implications of the present findings, the SUCLG1-POLRMT axis should be tested in oncogenic settings associated with tyrosine kinase mutations beyond FLT3-ITD and with mutations in tricarboxylic acid cycle enzymes, notably isocitrate dehydrogenase (IDH) and succinate dehydrogenase (SDH) genes. The status of additional posttranslational modifications (e.g., phosphorylation) on POLRMT upon modulation of succinylation levels should also be investigated.

Additionally, targeting SIRT5 with pharmacological inhibitors represents a significant therapeutic strategy within this system. However, SIRT5 plays different enzymatic roles, such as demalonylase and deglutarylase, in addition to desuccinylase (Du et al, 2011), and affects multiple substrates. Thus, its enzymatic specificity towards POLRMT compared to other substrates in the context of AML warrants further investigation. The positive correlation of SUCLG1 expression with electron transport chain (ETC) components across numerous cancers suggests that SUCLG1 and SIRT5 influence POLRMT activity and mitochondrial biogenesis in non-AML cancers. Although FLT3 wild-type and mutant cancers present with comparable sensitivity to venetoclax (Konopleva et al, 2022), the levels and activity of SUCLG1, SIRT5, and POLRMT and the potential synergy between venetoclax and SIRT5 inhibitors should be further examined in FL3-IDT AML cases. Lastly, exploring both TCA-dependent and independent pathways that could compensate for or confer resistance to SIRT5 inhibitors will be critical.

In summary, these research findings provide novel insights into how FLT3 mutations contribute to the altered mitochondrial biogenesis characteristic of AML progression through their effect on SUCLG1 activation and subsequent decrease in POLRMT succinylation, pinpointing potential therapeutic targets. Protein succinylation is a dynamic modification, and the TCA cycle intermediate succinyl-CoA functions as a signaling molecule that modifies and negatively regulates POLRMT, impairing POLRMT binding to mtDNA.
